# Neuroimmunology and ageing – the state of the art

**DOI:** 10.1186/s12979-024-00411-w

**Published:** 2024-01-10

**Authors:** Moisés E. Bauer, Graham Pawelec, Roberto Paganelli

**Affiliations:** 1 Laboratory of Immunobiology, School of Health and Life Sciences, Pontifical Catholic University of Rio Grande do Sul (PUCRS), Porto Alegre, Brazil; 2 Department of Immunology, University of Tübingen, Tübingen, Germany; 3 Health Sciences North Research Institute, Sudbury, ON Canada; 4 YDA-Institute of Clinical Immunotherapy and Advanced Biological Treatments, Pescara, 65121 Italy; 5 Internal Medicine, UniCamillus, International Medical University in Rome, via di Sant’Alessandro 6, Rome, 00131 Italy

## Background

For decades, the central nervous system (CNS) was known as an immune-privileged site. This concept was formulated based on experimental studies that demonstrated that, unlike what was observed in peripheral organs, skin grafts were not rejected when transplanted into the brain parenchyma. The presence of the blood-brain barrier (BBB), capable of selectively regulating the entry of molecules and cells from the bloodstream into the brain parenchyma, and the absence of conventional lymphatic vessels contributed to reinforcing this concept [1]. However, meningeal lymphatic vessels in the dura mater and, more recently, glymphatic vessels have raised questions about the dogma of the CNS as an immune-privileged site [2, 3]. The CNS has thus adopted mechanisms that enable communication with the immune system, which is crucial for a healthy brain.

Recent studies highlight the borders of the CNS as pivotal sites of neuro-immune interactions. Under physiological conditions, characterized by the absence of leukocytes in the brain parenchyma, innate immune cells, such as macrophages, and adaptive immune cells, such as T and B cells, are present in meningeal regions, in the choroid plexus and perivascular spaces. In addition to actively participating in immune surveillance in the CNS, these cells contribute to the maintenance of brain homeostasis and may influence behavioural and cognitive responses [4, 5]. For instance, cytokines secreted by immune cells, localized at the brain borders, may change behaviour through modulation of neuronal activities in distinct brain regions [5]. Indeed, several T cell-related cytokines have been shown to modulate complex CNS functions, by inducing changes in neuronal physiology: interferon γ (IFN-γ) alters sociability [6], IL-17 maintains anxiety and spatial learning [7], and IL-4 regulates learning and memory [8].

Inflammaging is a low level pro-inflammatory state which is believed to be a major contributor to biological aging which underlies many age-associated diseases. Peripheral inflammation significantly affects brain function and contributes to the development of several neurological disorders. Changes in interactions between the CNS and the immune system, such as those observed during ageing, could predispose to the development of neurodegenerative and neuropsychiatric diseases. Both ageing and neuropsychiatric disorders of older adults seem to converge on the pathogenetic role of inflammation, hence the notion of neuroinflammation. Increasing evidence indicates the role of neuroinflammation in age-related neurodegenerative diseases, such as Alzheimer’s Disease (AD) and Parkinson’s disease (PD). The neuropathological features of these diseases include aggregation and accumulation of intracellular and/or extracellular proteins that are associated with neuronal loss in specific regions of the brain. Furthermore, proliferation and activation of glial cells (i.e., “gliosis”) are well established in these diseases. In this context, neuroinflammation is essentially characterized by the activation of microglia and innate inflammatory pathways, such as activation of the NLRP3 inflammasome, increased expression of transcription factors for genes encoding inflammatory proteins (e.g., nuclear transcription factor κ-B, NF-κB), increased secretion of pro-inflammatory cytokines and chemokines, and increased production of reactive oxygen species (ROS) [9]. In addition, the phagocytic clearance of protein aggregates and cellular debris by microglia deteriorates during ageing and degenerative disorders [10]. Indeed, much of the literature points to an important role of tissue phagocytic cells in the pathogenesis of AD. On the other hand, peripheral immune cells, such as lymphocytes, may have modulatory functions, but the contribution of these cells to the pathogenesis of neurodegenerative diseases is still debatable [11]. Recent additions to this scenario have been published with the transcriptomic atlas of different regions of the brain [12].

## The topical collection

This Topical Collection of papers on Neuroimmunology and Ageing showcases a growing body of work characterizing the complex crosstalk of brain and immune cells (CNS-resident or circulating) during healthy ageing as well as characterizing the neuroinflammation observed in neurodegenerative diseases and neuropsychiatric disorders.

### Neuroimmune changes during healthy brain ageing: impact of sex and lifestyle

As the brain ages, the immune cells at peripheral sites as well as in the CNS change significantly – and T cells from outside the brain may infiltrate the brain tissue. The reasons for this age-related trafficking of T cells into the brain are not well understood. Several papers in this Topical Collection contribute to clarifying this issue. Analysing the transcriptional profiles of individual cells from young and old mice, ZHANG et al. (2022) constructed interaction networks between brain endothelial cells (BECs), microglia and T cells (https://immunityageing.biomedcentral.com/articles/; 10.1186/s12979-022-00289-6). T-cell infiltration was observed in the subventricular zone of aged mice. Cell–cell interaction analysis revealed that aged microglia released CCL3 to recruit peripheral CD8 + memory T cells. As a potential consequence, the aged microglia changed their phenotype towards a pro-inflammatory state, releasing TNF-α to upregulate the expression of VCAM1 and ICAM1 in BEC - therefore promoting the migration of peripheral T cells into the brain. In vitro, experiments revealed that human microglia would also transit to a chemotactic phenotype when treated with CSF from elderly subjects. This study reveals the neuroimmune pathways involved in maintaining brain homeostasis during normal ageing. A unique map of immune cells in the “normal” CNS is a useful guide to assess the changes observed in disease. This was provided by NEVALAINEN et al. (2022) in a study of 22 cell types, representative of natural and adaptive immune cells, identified in 13 different brain regions of 55 donors without diseases affecting the brain. The proportion of immune cells was determined by assessing multiple gene signatures and analyzed by the cytometry tool CIBERSORTx, thus establishing the cell subtypes using levels of expression of 547 signature genes (10.1186/s12979-022-00302-y). The effect of aging was to increase the presence of innate immune cells (mainly monocytes) and decrease all adaptive immune cells in distinctive brain regions. The age-associated differences in the composition of infiltrating immune cells are consistent with a role in tissue homeostasis.

Women with major depression tend to experience more cognitive problems than men. This sex-related cognitive bias includes negative thinking patterns that affect how people remember and interpret information. The cognitive bias is influenced by neurogenesis in the hippocampus and by neuroinflammation. Given the association of cognitive bias to neurogenesis and inflammation, HODGES et al. (2022) examined associations between cognitive bias, neurogenesis in the hippocampus, and cytokine levels in the ventral hippocampus (HPC) and basolateral amygdala (BLA) of male and female rats across the lifespan (https://immunityageing.biomedcentral.com/articles/; 10.1186/s12979-022-00299-4). Following testing for cognitive bias, male rats had more inflammatory cytokines in the ventral HPC than females in adolescence. In young adulthood, female rats had more IFN-γ, IL-1β, IL-4, IL-5, and IL-10 in the BLA than males. Middle-aged rats had more IL-13, TNF-α, and CXCL1 in both regions than younger groups. Adolescent male rats had higher hippocampal neurogenesis than adolescent females after cognitive bias testing. Neurogenesis in the dorsal HPC was negatively associated with negative cognitive bias in young adult males. Taken together, these results document that the association between negative cognitive bias, hippocampal neurogenesis, and inflammation in the brain differs by age and sex.

Age-related changes in lifestyle (for example, those impacting body composition) may also be associated with neuroinflammation and increased morbidity risk. Obesity rates are rising significantly around the world. Obesity leads to many complications, such as increasing the risk of cognitive decline in older age. Several changes in the immune system, such as inflammageing and immunosenescence, are common in both obesity and ageing and may affect cognitive decline. Therefore, immune system changes across the lifespan may influence how obesity affects neuroinflammation and cognitive decline related to it. To better understand this relationship, HENN et al. (2022) investigated the metabolic and inflammatory profiles associated with cognitive changes using a mouse model of obesity with a high-fat diet (HFD) (https://immunityageing.biomedcentral.com/articles/; 10.1186/s12979-022-00323-7). Mice on an HFD had significant age-related changes in hippocampal gene expression. The HFD caused a dysmetabolic phenotype in both young and middle-aged groups. However, older age exacerbated HFD cognitive and neuroinflammatory changes, with altered expression of hippocampal inflammatory genes. Taken together, these data indicate that obesity (HFD) promotes a premature ageing phenotype in the brain, which is indicative of inflammageing and immunosenescence. The pro- or anti-inflammatory effects of diet on cognitive impairment were assessed by LIU et al. (2023) in a cohort of 2944 people followed for 2 years; the longitudinal study showed increased decline in males with a higher dietary inflammatory index. This was further confirmed by a nested case-control study. The relationship also held with systemic inflammatory indexes derived from leukocyte counts, in agreement with an array of inflammatory cytokines, and it was mediated by one of them (SIRI) which acted upon leukocyte telomere length as well as mitochondrial DNA copy number, which were also independently associated with mild cognitive impairment (10.1186/s12979-022-00326-4).

### Neuroimmune changes associated with neuropsychiatric disorders

Neuropsychiatric disorders, including major depression and bipolar disorder, have been associated with several characteristics of premature ageing, such as chronic low-grade inflammation (inflammageing), cells with shortened telomeres and dysregulated immune responses [13]. These changes may impact disease progression (ex., promoting neuroinflammation) as well as immunity to infections and vaccines. Here, FORD and SAVITZ (2022) review recent data suggesting that depression is a risk factor for both adverse outcomes following COVID-19 infection and for reduced COVID-19 vaccine immunogenicity (https://immunityageing.biomedcentral.com/articles/; 10.1186/s12979-022-00288-7).

In an epidemiological study of very old Chinese adults in Hainan, SUN et al. (2022) found that depressive symptoms, assessed according to the Geriatric Depression Scale, were present in women of more advanced age (99 yrs average), and were associated with higher IgA serum levels, together with smaller but significant decreases in haemoglobin, IgM and C3 levels (10.1186/s12979-022-00283-y). This finding deserves further scrutiny for a mechanistic explanation.

### Neuroinflammation in the pathogenesis of neurodegenerative diseases

Neuroinflammation is also key to the pathogenesis of neurodegenerative diseases [10]. Natural antibodies (nAbs) against aggregation-prone proteins have been found in both healthy subjects as well as in patients with neurodegenerative disorders. Here, PAGANELLI et al. (2023) measured nAbs to Aβ protein in a group of Italian patients with Alzheimer’s Disease (AD), vascular dementia, non-demented patients with Parkinson’s Disease (PD) and healthy elderly controls (https://immunityageing.biomedcentral.com/articles/10.1186/s12979-023-00336-w). The Aβ antibody levels in AD were similar to age- and sex-matched controls, but significantly reduced in PD. This study may identify patients that could be more prone to amyloid aggregation.

Peripheral evidence of inflammatory processes may shed light on localized neuroinflammation, as COSTANTINI et al. (2023) propose in their paper on different gene expression of acetylcholine receptor (AChR) subunits in blood mononuclear cells (10.1186/s12979-023-00329-9). Some types were underrepresented in leukocytes from patients with Lewy body-type dementia and Alzheimer disease, but with different patterns, as also the case for inflammatory cytokines which were more highly expressed but at different levels and without association with AChRs – despite reports that AChR7 and AChR2/4 suppress the release of proinflammatory cytokines when activated.

The blood-brain interaction was also investigated by LI et al. (2023) in mice transgenic for APP/PS1, and in a parabiotic model with wild-type mice. They studied the effects of high levels of amyloid Aβ on circulating macrophages (10.1186/s12979-023-00366-4) and found that high plasma levels of Aβ1–42 play a biphasic role, i.e. inhibiting effects on peripheral pro-inflammatory macrophages in the early stage of the model, but promoting inflammation in the late stage. The effects are suggested to be mediated by myeloid-derived suppressor cells in the spleen and myeloid precursors in the bone marrow, adding more layers of complexity.

Signatures of pain arising from nerve injuries are generally associated with changes in cell numbers, trafficking, and immune phenotypes within dorsal root ganglia (DRG). In this context, CHOCONTA et al. (2023) investigated neuroimmune processes involved in the DRG of Fabry disease (https://immunityageing.biomedcentral.com/articles/10.1186/s12979-023-00346-8). Pain in Fabry disease (FD) is generally accepted to result from neuronal damage in the peripheral nervous system because of excess lipid storage caused by alpha-galactosidase A (α-Gal A) deficiency. Using a mouse model of FD, they report significant alterations of lysosomal signatures in sensory neurons and of macrophage morphology and phenotypes in the DRG. The changes in macrophage morphology are suggestive of premature ageing, and these cells could be targeted in better therapies.

### Interventions to mitigate age-related neuroinflammation

This Topical Collection includes articles exploring the beneficial effects of interventions aimed to mitigate neuroinflammation. GONG et al. (2023) investigated the molecular mechanisms by which glycyrrhizic acid (GA), a saponin compound often used as a flavouring agent, improves cognition in mice through immunomodulation (https://immunityageing.biomedcentral.com/articles/10.1186/s12979-023-00337-9). Single-cell sequencing data of PBMCs revealed that GA reduced age-related increases in myeloid cells and increased numbers of lymphoid lineage subpopulations. In vitro, GA significantly promoted the differentiation of hematopoietic stem cells toward lymphoid lineages, of note CD8^+^ T cells. Moreover, GA inhibited the differentiation of CD4^+^ T cells and myeloid (CD11b^+^) cells by binding to S100 calcium-binding protein 8 (S100A8) protein.

The mechanism of beneficial effects of exercise on the brain was investigated by HAN et al. (2023) by exploring the phenotypic and functional changes induced in microglia by the metabolite product of exercise, lactate. Activated microglia exhibit abnormal morphology and proliferation and release inflammatory and bioactive molecules, which may damage neurons; these investigators found that addition of lactate, through a lactate “timer”, accelerated the upregulation of homeostatic genes producing an anti-inflammatory reparative phenotype transition of microglia in mice pretreated with aluminium-D-Galactose which induces an Alzheimer-like syndrome. Induction of Arginase-1 reflected the initiation of a reparative phenotype through lactylation of histone H3 in brains of mice after exercise training, which also demonstrated improved cognitive function and less neuronal loss in the brain.

ZAMORANO et al. (2023) showed that treatment of 9-month-old male mice with the chemotherapeutic agent cisplatin causes cognitive defects that are associated with the formation of tau deposits in the hippocampus. Nasal administration of mesenchymal stromal cells (MSC) at 48 and 96 h after cisplatin prevented formation of tau deposits and normalized syndecan-2 and GFAP expression (10.1186/s12979-023-00328-w). Cisplatin-induced tau cluster formation was associated with reduced executive functioning and working/spatial memory; nasal administration of MSC prevented these cognitive defects. Notably, delayed MSC administration (one month after cisplatin) also prevented tau cluster formation and cognitive alterations. Developing nasal MSC administration for treatment of accelerated brain aging and cognitive problems in cancer survivors should be feasible and would greatly improve their quality of life.

Neuroinflammation also has a key role in infection-induced neurological injury, particularly in older adults with increased incidence of sepsis following CNS infection. Brain tissue-resident memory T cells (bT_RM_) are recruited during CNS infection and promote pathogen control as well as deleterious inflammation. Here, CASSIDY et al. (2022) explored the anti-inflammatory actions of the microRNA miR-155 during neuroinvasive infection of mice with *Listeria monocytogenes* (https://immunityageing.biomedcentral.com/articles/; 10.1186/s12979-022-00281-0). Notably, anti-miR-155 treatment reduced the accumulation of brain myeloid cells in aged mice after infection, whereas CD8^+^ bT_RM_ were unaffected.

In a transgenic RNAi screen using Drosophila as a model, YUE et al. (2023) report that knockdown of Dsor1 (the Drosophila MAPK kinase dMEK) suppressed protein inclusion containing TAR DNA-binding protein of 43 kDa (TDP-43) toxicity, without altering TDP-43 phosphorylation or protein levels (10.1186/s12979-023-00354-8). TDP-43 is an important DNA/RNA-binding protein that is associated with age-related neurodegenerative diseases such as amyotrophic lateral sclerosis and frontotemporal dementia. Further investigation revealed that the Dsor1 downstream gene rl (dERK) was abnormally upregulated in TDP-43 flies, and neuronal overexpression of dERK induced profound upregulation of antimicrobial peptides (AMPs). This induced immune overactivation in TDP-43 flies, which was suppressed by downregulation of the MEK/ERK pathway in TDP-43 fly neurons. Neuronal knockdown of Dnr1, a negative regulator of the Drosophila immune deficiency (IMD) pathway, activated innate immunity and boosted AMP expression independent of regulation by the MEK/ERK pathway. An FDA-approved MEK inhibitor (trametinib) markedly suppressed immune overactivation, alleviated motor deficits and prolonged the lifespan of TDP-43 flies, but did not exhibit such features in either AD or spinocerebellar ataxia type 3 (SCA3) fly models. This study sheds new light on the pathogenesis of TDP-43 neurological damage.

MÜLLER and DI BENEDETTO (2023) review neuroimmune interactions in the aged brain and highlight the impact of COVID-19 on the functional systems already modulated by immunosenescence and neuroinflammation (https://immunityageing.biomedcentral.com/articles/10.1186/s12979-023-00341-z). They discuss the potential neuroimmune interactions involved in COVID-19 sequelae, reviewing mechanisms and biological factors that may contribute to persisting long COVID conditions. The main biological mechanisms involved with long COVID are discussed as well as various interventional options (e.g. nutritional, exercise and behavioural) that may mitigate the neuroimmune activation associated with unbalanced inflammatory responses.

In a comprehensive review, SAHEBNASAGH et al. (2022) describe the concept of neurohormesis and apply it to herbal remedies and plant-derived drugs used to alleviate or cure the symptoms of neurological diseases (10.1186/s12979-022-00292-x). These include both neurodegenerative (Alzheimer, Parkinson) as well as other neurological disorders such as autism and Huntington’s disease. The reader is taken through neurohormetic herbal medicines (i.e. the hormetic properties of phytochemicals used to treat neurological disorders*)* for aging, neuroprotection, memory enhancement, and the effects of these phytochemicals on the immune system and the mitochondria functions.

## Conclusions

The studies described in the 17 papers in this Topical Collection give an all-encompassing view of the complex relationships between immune system changes with ageing, and several diseases of the central nervous system, as well as novel potential targets able to influence the progression of neurological impairment.

## Data Availability

Not applicable.
